# Advancing equity in perinatal PTSD: a systemic trauma framework for written exposure therapy

**DOI:** 10.3389/fpsyt.2026.1871794

**Published:** 2026-08-26

**Authors:** Tiffany R. Williams, Joanne K. Daggy, David M. Haas

**Affiliations:** 1 Department of Psychiatry, Indiana University School of Medicine, Indianapolis IN, United States; 2 Department of Biostatistics and Health Data Science, Indiana University School of Medicine, The Richard M. Fairbanks School of Public Health, Indianapolis IN, United States; 3 Department of Obstetrics and Gynecology, Indiana University School of Medicine, Indianapolis IN, United States

**Keywords:** brief interventions, maternal morbidity, maternal–infant bonding, obstetric complications, perinatal PTSD, systemic trauma, trauma−focused treatment, written exposure therapy (WET)

## Abstract

This article applies a systemic trauma framework to perinatal PTSD and advances Written Exposure Therapy as a scalable, equity-aligned intervention that can be integrated into routine perinatal care pathways, addressing critical gaps in current obstetric and mental health systems. Perinatal posttraumatic stress disorder (PTSD) affects ~9% of pregnant and postpartum individuals in the United States, with another 18% at elevated risk, yet it remains inconsistently identified in clinic care. From a systemic trauma perspective, perinatal PTSD arises from interactions between individual experiences and structural inequities in healthcare, including discrimination, fragmented services, and access barriers. These factors intersect with pregnancy-, birth-, and NICU-related stressors to increase maternal morbidity, disrupt maternal–infant bonding, and affect early developmental outcomes. Despite this burden, few trauma-focused treatments are feasible during the perinatal period, as standard protocols often conflict with caregiving and medical demands. Written Exposure Therapy (WET), a five-session intervention, promotes trauma recovery through repeated written engagement with trauma narratives, facilitating emotional processing and extinction of trauma-related distress. WET offers a scalable alternative, with preliminary evidence for obstetric groups comparable to longer treatments in non-perinatal populations. This conceptual synthesis advances a systemic trauma-informed framework for embedding WET within perinatal care pathways, emphasizing how brief, low-burden interventions can be integrated into routine obstetric, postpartum, and NICU-linked settings to improve access to trauma-focused care. By linking mechanisms of trauma recovery with health system delivery structures, this framework clarifies how scalable interventions like WET may reduce inequities in treatment access and improve maternal and infant outcomes. While the proposed framework is grounded primarily in evidence from U.S. healthcare systems, it provides a foundation for future adaptation and evaluation in diverse international perinatal care settings. Future priorities include testing feasibility, mechanisms, and equity-focused implementation across diverse perinatal populations.

## 1 Introduction

Perinatal posttraumatic stress disorder (PTSD) is increasingly recognized as a significant public health concern in the United States, affecting approximately 9% of perinatal individuals, with an additional 18% at elevated risk (1, 2). Emerging during pregnancy, triggered by childbirth, or persisting into the postpartum/Neonatal Intensive Care Unit (NICU) period, this condition is a consequential yet underrecognized driver of maternal morbidity, infant complications, and rising health system costs (3, 4). The condition involves both the emergence of new symptoms in response to perinatal events (e.g., medical complications, traumatic birth, NICU stress) and the exacerbation of pre-existing trauma histories (5). Untreated perinatal PTSD is linked to adverse obstetric outcomes, increased likelihood of NICU exposure, and elevated infant socioemotional risk, yet screening and treatment remain inconsistent and frequently delayed. Individuals with trauma histories are approximately twice as likely to experience pregnancy-related mortality compared to those without such histories, with some groups (e.g., Black women) facing disproportionately elevated risk (6). These outcomes including preeclampsia, preterm birth, and low birth weight are leading contributors to pregnancy- and infant-related mortality (1, 2, 5, 7, 8).

PTSD not only increases the risk of adverse pregnancy outcomes (2, 9–12), it can also be intensified by those same complications, creating a reinforcing cycle of trauma exposure and physiological vulnerability (8, 13, 14). Across the perinatal period, targeted attention to evaluating and adapting evidence-based trauma treatments as early as possible is needed. Perinatal PTSD places both mother and infant at risk, contributing to impaired bonding, insecure attachment, and long-term threats to child health and development (11, 12). Despite the substantial clinical burden, early screening and delivery of evidence-based treatment remain limited. Thus, there is a persistent gap in scalable, low-burden interventions that reduce PTSD symptoms given its potential to prevent maternal and infant mortality (4). Perinatal PTSD is not a singular clinical entity and may reflect several distinct pathways. For some individuals, PTSD predates pregnancy and may be exacerbated by pregnancy- and birth-related stressors. For others, symptoms emerge during pregnancy in response to current medical, interpersonal, or systemic trauma exposures, whereas childbirth-related PTSD develops following traumatic labor, delivery, or associated complications. Although these pathways differ in etiology and timing, each may contribute to substantial maternal and infant morbidity and may benefit from timely trauma-focused intervention (2, 5, 14, 15).

A central gap persists: perinatal individuals are chronically underrepresented in trials of gold-standard PTSD interventions, and standard multi-month therapies are poorly suited to the realities of pregnancy and early postpartum life including frequent medical visits, sleep disruption, and childcare demands (15–18). The field requires brief, accessible, trauma-focused treatments that can be feasibly embedded throughout the perinatal continuum, rather than delayed until after birth. Written Exposure Therapy (WET) is a brief standardized, protocol-driven exposure-based treatment that offers a pragmatic, scalable option to reduce symptom burden, support maternal–infant bonding, and potentially interrupt intergenerational transmission of trauma when integrated into obstetric pathways (12, 19–21).

A systemic trauma lens clarifies how perinatal PTSD emerges not only from discrete medical events but from the broader conditions in which pregnancy and birth occur (20). In ongoing program development, a WET protocol is being piloted virtually embedded within perinatal care pathways to address persistent barriers such as time constraints, access limitations, and childcare demands. These barriers are not isolated challenges but manifestations of broader systemic conditions that fundamentally shape perinatal PTSD risk and recovery.

Taken together, these gaps underscore a central challenge: perinatal PTSD is both highly consequential and poorly addressed by existing treatment models that are not designed for the structural, temporal, and caregiving realities of pregnancy and early postpartum life. This mismatch highlights the need for brief, trauma-focused interventions that can be feasibly embedded within perinatal care pathways, positioning WET as a promising and scalable solution.

## 2 Conceptual approach

The current manuscript is a conceptual synthesis informed by a targeted review of literature on perinatal PTSD, systemic trauma, and trauma-focused treatments. Relevant studies and frameworks were identified through searches of PubMed, PsycINFO, and Google Scholar, focusing on peer-reviewed English-language publications from 2010 to 2026. Search terms included combinations of “perinatal,” “pregnancy,” “postpartum,” “PTSD,” “trauma,” and “written exposure therapy.” Priority was given to recent empirical studies (2023–2026), foundational theoretical work on systemic trauma, and emerging clinical and feasibility studies of WET in high-risk or perinatal populations. Non-peer-reviewed sources and case reports were excluded. This approach was selected to integrate theoretical and clinical evidence and develop a clinically relevant, equity-focused framework for perinatal psychiatry. Studies were selected based on their relevance to perinatal PTSD, trauma mechanisms, systemic factors influencing care, and the feasibility or implementation of trauma-focused interventions within perinatal settings.

Consistent with the aims of a conceptual synthesis, studies were not weighted according to a formal evidence hierarchy. Rather, publications were prioritized based on their relevance to perinatal PTSD, systemic trauma theory, and the clinical feasibility or implementation of Written Exposure Therapy. Recent studies (2023–2026) were emphasized to reflect current developments in perinatal mental health, while foundational studies were included when they established the theoretical or empirical basis of key concepts, including the efficacy and mechanisms of WET.

Thematic integration was conducted using a hybrid deductive–inductive approach. Initial domains were guided by systemic trauma theory, including structural inequities, care fragmentation, and contextual barriers to treatment engagement. These domains were then refined through iterative review of the literature, allowing key clinical and implementation-relevant themes (e.g., feasibility constraints, access barriers, and integration within care pathways) to emerge and be organized into the proposed framework. These thematic domains informed the organization of the conceptual framework and guided the identification of key clinical and implementation pathways presented in subsequent sections. Themes were refined through iterative review of the literature and evaluated for consistency with the proposed systemic trauma framework and their relevance to perinatal clinical care and implementation.

## 3 Systemic trauma and perinatal vulnerability

Systemic trauma extends beyond individual events to encompass the institutional, cultural, and structural forces that shape exposure to harm and access to care during the perinatal period. In the context of perinatal health, systemic trauma reflects inequitable healthcare practices, discriminatory treatment, limited access to services, and sociopolitical conditions that heighten vulnerability (22). These contextual pressures shape how trauma is conceptualized and experienced, how symptoms develop, how easily individuals can access support, and how trauma is treated (23, 24). These same systemic forces also shape infant outcomes, as the structural and sociocultural conditions that heighten perinatal PTSD risk simultaneously influence early bonding, caregiving capacity, and the developmental environments into which infants are born (22).

Within perinatal care specifically, obstetric racism, biased clinical encounters, disrupted continuity of care, and barriers related to transportation, childcare, insurance, or clinician availability can compound distress and restrict treatment engagement (25–28). Trauma can also arise indirectly through repeated exposure to distressing birth stories, community experiences of loss, or the chronic stress of navigating unsafe or invalidating systems (29–32). Understanding these systemic forces is essential because they influence outcomes long before and long after a single obstetric event. These systemic pressures intersect with pregnancy-specific stressors, such as hypertensive disorders, preterm labor, fetal health concerns, unanticipated interventions, and discriminatory OB experiences, all which can elevate PTSD risk and complicate care engagement (8, 10, 13, 29). For marginalized birthing people, brief, accessible, low-barrier trauma focused care is therefore an equity strategy, not merely a clinical convenience (18, 33, 34).

A systemic trauma framework clarifies the complexity of perinatal PTSD, illuminates why maternal and infant mortality persist, and reshapes how the field understands both risk and intervention (22). Rather than viewing trauma-focused, evidence-based treatments—traditionally delivered through referral-based, multi-session outpatient psychotherapy—as optional or “non-traditional,” a systemic trauma lens positions these interventions as essential components of perinatal care that can directly reduce clinically significant morbidity (2, 18). By making structural, sociocultural, and logistical barriers visible, this lens alerts clinicians and health systems to persistent forces that undermine safety, choice, and engagement in perinatal care. It also accentuates why brief, flexible, low-burden treatments must be embedded directly within obstetric pathways: without addressing the systemic conditions that constrain access, even the most effective interventions cannot be equitably delivered or sustained (17, 22). Operating within a systemic trauma framework highlights why scalable trauma-focused treatments, such as WET, are essential to counteract the forces that otherwise limit recovery (35).

Applying a systemic trauma lens to the conceptualization, treatment, and evaluation of perinatal PTSD strengthens outcomes for both parents and infants because it directly addresses the structural, sociocultural, contextual, and pregnancy specific factors that shape the development and persistence of PTSD. This lens makes these intersecting drivers visible and clarifies how they contribute to preventable morbidity and mortality for parents and babies when they are not addressed in care. Without a systemic trauma perspective, these forces remain overlooked, and trauma focused interventions are less likely to be delivered effectively or equitably (17).

**Figure 1:**
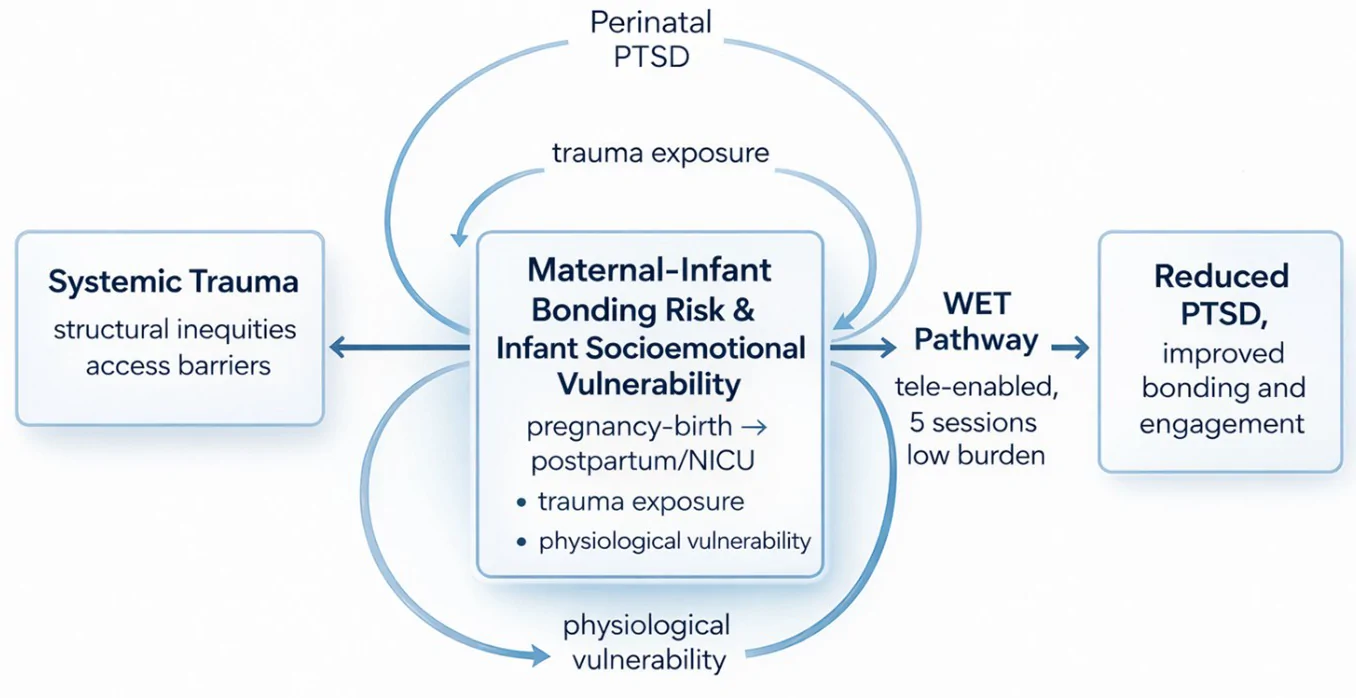
System map of perinatal PTSD within systemic trauma and the role of written exposure therapy.

**Figure 1** illustrates how perinatal PTSD arises and is maintained within intersecting systemic, physiological, and relational contexts during pregnancy, childbirth, and the postpartum period. Structural conditions, including healthcare access barriers and broader inequities, contribute to trauma exposure and heightened physiological vulnerability during pregnancy and perinatal hospitalization, including NICU admission. These conditions increase risk for maternal PTSD and disrupt maternal–infant bonding and contribute to increased infant developmental risk. Bidirectional pathways depict how trauma exposure and physiological stress mutually reinforce perinatal PTSD and relational risk within a conceptual framework. The WET pathway is shown as a brief, low-burden, tele-enabled intervention designed to be embedded within obstetric care. By intervening within the care pathway itself, WET is hypothesized to potentially reduce PTSD symptoms, improve maternal engagement and bonding, and interrupt downstream effects on infant vulnerability, addressing trauma within the systemic conditions in which it occurs.

Within this framework, WET is hypothesized to act primarily at the level of trauma processing by reducing avoidance, facilitating emotional engagement with trauma memories, and decreasing PTSD symptom severity (19, 36, 37). Improvements in PTSD symptoms may enhance maternal functioning, engagement in care, and maternal-infant bonding, thereby disrupting downstream pathways linking trauma exposure to adverse maternal and infant outcomes (11, 12, 38, 39). WET does not directly modify the structural conditions that contribute to systemic trauma; rather, it mitigates their clinical consequences by increasing access to evidence-based trauma treatment within existing healthcare systems (15, 17, 18, 33, 34).

## 4 The evidence gap across the perinatal timeline

Perinatal PTSD remains insufficiently addressed within routine obstetric care, despite affecting an estimated 10–19% of pregnant individuals (1, 40). Although the disorder carries substantial social and health implications for both parent and infant, very few interventions are designed to treat PTSD during pregnancy or the early postpartum period (4, 17, 18, 38). Most available research focuses narrowly on childbirth-related PTSD, leaving the broader perinatal trajectory, prenatal onset, antenatal exacerbation, and postpartum persistence, largely unexamined. As a result, perinatal populations are rarely included in trials of exposure-based PTSD treatments, limiting the development of tailored recommendations and restricting clinicians’ ability to make evidence-aligned decisions. Earlier identification and intervention could meaningfully reduce distress and improve relational outcomes, but the field still lacks treatments that are both effective and feasible for individuals navigating the systemic, structural, and logistical constraints that shape perinatal trauma exposure and access to care (2).

Pregnancy-related trauma histories and obstetric complications increase risk for perinatal PTSD and are associated with preterm birth, low birth weight, neonatal complications, and higher NICU admissions, likely mediated through chronic stress physiology (1, 2, 8, 14, 31, 41). Unplanned surgical deliveries, hemorrhage, peritraumatic dissociation, and negative obstetric interactions during childbirth can further precipitate or intensify PTSD symptoms (1, 4, 14, 35). In the postpartum and NICU context, core PTSD features such as numbing, hyperarousal, and avoidance can impair bonding, disrupt dyadic synchrony, and undermine sensitive responses to infant cues, contributing to downstream socioemotional and neurodevelopmental effects (38, 39, 42). NICU exposure may extend parental symptoms for months (15, 36).

Gold-standard PTSD treatments such as Cognitive Behavioral Therapy (15, 36, 40) (CBT), Prolonged Therapy (PE) and Cognitive Processing Therapy (CPT) have historically overlooked the needs of perinatal individuals, reflecting broader patterns of underrepresentation in trauma research (18, 43). Even when available, these 8–12+ session protocols are often misaligned with perinatal realities, as caregiving demands, medical complexity, and structural mistrust make it difficult to initiate and sustain extended courses of care (18, 33). These logistical and systemic barriers disproportionately affect those already at highest risk, such individuals navigating inequitable healthcare access, discrimination, transportation limitations, or disrupted continuity of care, thereby widening disparities in who receives evidence-based PTSD treatment (15, 44). In response, scholars have called for brief, scalable approaches that address symptoms in high-risk perinatal and postpartum populations and that can be initiated as early as possible during pregnancy or after birth (18). Collectively, these limitations point to the need for interventions that can be delivered within the structural, temporal, and caregiving constraints of perinatal life.

## 5 Findings from literature and conceptual integration

### 5.1 Prevalence and clinical burden

The literature consistently identifies perinatal PTSD as a significant maternal mental health concern associated with increased maternal morbidity, obstetric complications, and elevated risk for adverse pregnancy outcomes (2, 40). Literature consistently links perinatal PTSD to increased maternal morbidity, including obstetric complications and heightened risk for adverse pregnancy outcomes. It is also associated with disruptions in maternal–infant bonding and early socioemotional development, positioning perinatal PTSD as both a mental health and public health concern (10, 12, 13, 38).

### 5.2 Systemic drivers of perinatal PTSD

Findings indicate that perinatal PTSD is shaped by systemic conditions beyond individual trauma exposure (22–24). Recurring themes include obstetric racism, fragmented care systems, and structural barriers to accessing mental health treatment (27, 29, 30, 45). These factors increase trauma exposure while simultaneously limiting engagement in care (15, 17, 33).

### 5.3 Gaps in the existing evidence base

The literature reveals persistent limitations that constrain clinical translation. Perinatal populations remain underrepresented in PTSD trials (15, 17, 18), and standard treatments are often misaligned with perinatal realities due to their length and intensity (15, 16). Few studies examine subgroup variation, limiting insight into disparities (29, 30, 45), and findings are frequently aggregated across the perinatal timeline, obscuring differences between prenatal, intrapartum, postpartum, and NICU contexts (15, 36, 40). The available literature is also disproportionately U.S.-centric, with relatively limited global data on perinatal PTSD prevalence and trauma-focused treatment feasibility, particularly in low- and middle-resource settings (2, 18). This constrains the generalizability of current findings across diverse healthcare systems.

### 5.4 Emerging evidence for written exposure therapy

WET demonstrates comparable efficacy to gold-standard PTSD treatments in randomized trials while requiring fewer sessions (36, 37, 46, 47). In randomized clinical trials, WET has demonstrated noninferior reductions in PTSD symptom severity compared with both PE and CPT. For example, both treatments produced substantial reductions in PTSD symptoms over time, with CAPS-5 scores remaining comparable between WET and PE across follow-up assessments through 30 weeks, supporting noninferiority despite the shorter treatment format (46). Similarly, WET produced reductions in PTSD symptoms comparable to CPT while demonstrating substantially lower treatment dropout during active treatment (6.3% vs. 31.7%) (48). Notably, WET has also been associated with lower treatment dropout rates than PE and CPT in several studies, suggesting potential advantages for populations facing substantial logistical and caregiving demands (46, 48). These findings are particularly relevant to perinatal populations, for whom competing medical appointments, childcare responsibilities, transportation barriers, and sleep disruption often limit engagement in longer treatment protocols.

Collectively, these findings suggest that WET may offer a particularly favorable balance of effectiveness and feasibility for populations facing major caregiving, medical, transportation, or scheduling barriers. Emerging feasibility studies further suggest that WET is acceptable and adaptable for high-risk and underserved populations, including perinatal groups, particularly when delivered via telehealth or integrated care models (20, 21, 49).

## 6 Clinical implications: why written exposure therapy fits the perinatal period

Building directly on the findings from the literature synthesis, WET aligns with four key domains identified as central to perinatal PTSD: clinical burden, systemic drivers, evidence gaps, and treatment feasibility. This alignment clarifies why WET is uniquely positioned as a scalable, equity-informed intervention within perinatal care systems. The core components underlying WET's feasibility and fit within perinatal care systems are summarized in **Box 1**.

Box 1 **Core components of written exposure therapy (WET)**

- Brief, time-limited protocol (typically five sessions) designed to minimize caregiver and clinician burden
- Structured written trauma narrative focused on an index traumatic experience
- Emphasis on emotional engagement and trauma memory processing, rather than cognitive restructuring or skills training
- Predictable session format that supports feasibility in medically complex, inpatient, or highly constrained care settings
- Minimal between-session demands, reducing reliance on assignments that may be difficult to sustain during the perinatal period and postpartum life
- Flexible delivery across in-person or telehealth modalities, enabling embedding within obstetric care pathways

### 6.1 Addressing clinical burden through time-limited, targeted intervention

The substantial prevalence and downstream consequences of perinatal PTSD highlight the need for interventions that can reduce symptom burden early and efficiently (2, 40). WET’s brief, five-session structure allows for rapid engagement and completion during pregnancy and the postpartum period, when individuals are navigating competing medical and caregiving demands (46, 49). By focusing directly on trauma memory processing, WET targets core PTSD symptoms that are associated with maternal morbidity, disrupted bonding, and infant socioemotional risk (10, 12, 39), offering a pragmatic pathway for early symptom reduction within existing care windows.

### 6.2 Responding to systemic drivers of perinatal PTSD

The literature emphasizes that perinatal PTSD is shaped by systemic factors, including inequitable care, discrimination, and fragmented service delivery (15, 17, 18, 30). WET’s structure directly mitigates these barriers by minimizing logistical and access-related burdens. Its predictable format, limited session count, and adaptability to telehealth reduce reliance on transportation, childcare, and extended clinic engagement, barriers that disproportionately affect marginalized populations (15, 33, 34). In this way, WET functions not only as a clinical intervention but also as an equity-responsive approach that can be embedded within the healthcare systems in which perinatal trauma occurs. Although telehealth may reduce several barriers to treatment engagement, it is not equally accessible to all perinatal populations. Digital barriers, including limited internet access, low digital literacy, privacy concerns, and lack of confidential home environments, may constrain participation for some individuals, particularly those already affected by structural disadvantage. Addressing these barriers will be important to ensure that telehealth-enabled delivery models advance, rather than inadvertently exacerbate, health inequities.

### 6.3 Bridging evidence gaps in perinatal PTSD treatment

Findings from the literature underscore that perinatal populations are underrepresented in trials of gold-standard PTSD treatments and that existing therapies are often poorly aligned with perinatal realities (16–19). WET addresses this gap by offering a model that retains core exposure-based mechanisms while reducing treatment duration and complexity. Emerging evidence from feasibility studies in high-risk and underserved populations suggests that WET can be adapted for use during pregnancy and postpartum, supporting its potential as a perinatal-specific application of evidence-based trauma treatment (20, 21, 49). This positions WET as a bridge between well-established PTSD treatment science and the unmet needs of perinatal populations.

### 6.4 Aligning with perinatal care delivery and feasibility constraints

The lack of differentiation across the perinatal timeline in existing research highlights the importance of interventions that can be flexibly delivered across pregnancy, childbirth, postpartum, and NICU contexts (15, 36, 40). WET is well suited to the perinatal continuum due to its low burden, minimal between-session demands, and adaptability across care settings. The intervention may be delivered during prenatal care, initiated during inpatient obstetric admissions, or integrated into postpartum and NICU-adjacent services (20, 21, 37). This flexibility supports continuity of trauma-focused care across transitions in the perinatal period and increases the likelihood of treatment completion within real-world clinical environments.

## 7 Clinical pathways for integrating WET across the perinatal continuum

Several limitations should be considered when interpreting this conceptual synthesis. First, this manuscript is not based on primary data but draws on secondary evidence and theoretical integration, which may be subject to selection and publication biases in the literature. Although we prioritized recent and peer-reviewed studies, the available evidence base reflects broader gaps in perinatal PTSD research, including limited representation of randomized controlled trials and underrepresentation of diverse perinatal populations. Second, the literature informing this framework is largely derived from U.S. and other high-income contexts, which may limit generalizability to low-resource or non-Western settings. Structural determinants of perinatal PTSD and access to trauma-focused care may differ substantially across global contexts. Finally, as a conceptual synthesis, this work cannot establish causal relationships but is intended to guide hypothesis generation, clinical application, and future empirical research.

Perinatal PTSD affects a substantial portion of pregnant and postpartum individuals, yet current clinical systems are not organized to identify or treat it effectively. Routine obstetric and pediatric settings rarely include trauma focused mental health pathways. This gap is magnified by systemic trauma forces such as inequitable care, fragmented services, and barriers related to transportation, childcare, and insurance, all which shape both the emergence of PTSD and the likelihood of receiving care. Given the well-documented links between perinatal PTSD, adverse obstetric outcomes, and disruptions in early bonding and infant socioemotional development, there is a pressing need for interventions that can be integrated into routine obstetric, pediatric, and NICU-adjacent care pathways.

WET offers such an opportunity, as it can be integrated into obstetric, pediatric, and NICU-adjacent pathways, allowing trauma-focused care to be delivered within settings that currently lack capacity for longer PTSD treatments. Because WET minimizes logistical, cognitive, and transportation demands, it is uniquely positioned to counteract the systemic barriers that limit access to evidence-based care for those most affected by perinatal trauma. Although WET can be embedded within obstetric care pathways, delivery would typically occur through trained behavioral health clinicians, including psychologists, social workers, counselors, or integrated behavioral health providers. Obstetric providers, nurses, and other members of the perinatal care team may play critical roles in screening, referral, engagement, and care coordination, rather than directly delivering the intervention. Integrated behavioral health models may represent the most feasible implementation strategy, allowing trauma-focused treatment to be delivered within existing perinatal care settings while maintaining appropriate behavioral health expertise and supervision.

Integrating WET into perinatal care requires coordinated efforts across the diverse disciplines that routinely engage with pregnant and postpartum individuals. Each professional group contributes uniquely to early identification, referral, engagement, and continuity of trauma-focused care, particularly within the systemic conditions that shape perinatal PTSD risk and recovery. **Table 1** outlines these role-specific clinical implications and practical applications, highlighting how obstetric, behavioral health, nursing, pediatric, lactation, home-visiting, community-based, and care-coordination professionals can support equitable and sustainable delivery of WET across the perinatal continuum. Successful integration of WET across perinatal settings will require role-appropriate training in trauma-informed screening, referral pathways, and interdisciplinary care coordination, as well as attention to common implementation barriers such as limited time, competing clinical responsibilities, and availability of behavioral health resources.

**Table 1:** Clinical implications and applications for WET across perinatal care roles.

Professional group	Clinical implications	Clinical applications
OB/GYN & Midwifery	Early identification of PTSD; normalization of trauma-focused referrals	Use brief screeners; co-schedule WET with prenatal visits; facilitate warm handoffs to IBH or psychiatry
Perinatal Mental Health Clinicians	Provide brief, structured trauma-focused care suited to pregnancy/postpartum demands	Deliver virtual or hybrid WET; coordinate with OB teams for medical safety and care continuity
Integrated Behavioral Health (IBH) Teams	Build sustainable trauma pathways within prenatal clinics	Establish referral processes; provide supervision and fidelity monitoring; track patient-reported outcomes
Social Workers, Doulas, Pediatrics/NICU	Identification of high-distress parents; support during NICU-linked trauma	Facilitate warm handoffs; reinforce psychoeducation about trauma and bonding; coordinate NICU-adjacent WET referrals
Lactation/Feeding Support Services	Recognize trauma-linked dysregulation during feeding interactions	Identify distress during feeding challenges; facilitate referral to mental health or IBH; reinforce trauma-informed engagement and care coordination
Home Visiting/Community Health Workers	Extend trauma-informed support to marginalized families postpartum	Identify symptoms in home environments; support tele-WET engagement; reinforce psychoeducation
Labor & Delivery Nurses	Recognize peritraumatic distress and early trauma indicators during birth	Initiate early identification and referral; flag patients for inpatient WET scheduling; reinforce trauma-informed communication and referral pathways
Primary Care/Family Medicine	Support continuity of care postpartum; additional PTSD screening opportunities	Integrate screening into well-parent and well-infant visits; link families to IBH for WET
Patient Navigators/Care Coordinators	Address systemic barriers such as transportation, insurance, and fragmented care	Support follow-through on referrals; coordinate transitions between prenatal, postpartum, and NICU settings

Building on these multidisciplinary roles, **Table 2** describes the clinical pathways through which WET can be embedded across prenatal, intrapartum, postpartum, and NICU-adjacent settings, emphasizing where and how the intervention can be delivered within existing care structures. To translate WET into routine clinical practice, perinatal care systems require clear, feasible pathways that align with existing structures rather than adding new burdens. **Table 2** below outlines a pragmatic, equity-aligned model for integrating WET across prenatal, intrapartum, postpartum, and NICU-related care settings. These pathways emphasize systematic screening, rapid referral, flexible delivery options, and sustainable implementation support allowing WET to be embedded directly into the clinical environments where perinatal PTSD can be most effectively identified and treated.

**Table 2:** Clinical pathways for integrating WET across the perinatal continuum.

Pathway component	Description	Rationale/goal
Screening Touchpoints	Pregnancy; intrapartum/before discharge; postpartum and NICU encounters	Identify PTSD early and reduce avoidance by establishing rapid referral routes.
Prenatal Clinics	Integrated Behavioral Health with warm handoffs from OB providers	Aligns WET with routine prenatal workflow; reduces attrition.
Perinatal Psychiatry Consults	Embedded psychiatric consults linked to OB/midwifery	Provides specialized support for high-risk or medically complex pregnancies.
Telehealth Delivery	Virtual WET during pregnancy and postpartum	Addresses mobility limits, transportation barriers, and scheduling constraints.
NICU-Adjacent Services	WET offered alongside NICU mental health support	Targets high-distress parents and mitigates downstream bonding disruptions.
Inpatient Obstetrics	Initiation or scheduling of WET during antepartum/postpartum admission	Captures high-risk patients who may otherwise be lost to follow-up.
FQHC/Safety-Net Settings	Delivery in Medicaid-focused or underserved clinics	Expands reach and advances equity in trauma treatment access.
Implementation Infrastructure	Referral pathways, tele-operations, safety checks, fidelity monitoring	Ensures quality, safety, and sustainability of WET integration.

## 8 Discussion

This conceptual synthesis highlights how WET functions as a trauma-focused intervention that is not only clinically feasible but structurally aligned with the conditions shaping perinatal PTSD. By mapping WET onto key domains identified in the literature, clinical burden, systemic drivers, evidence gaps, and feasibility constraints, this framework clarifies both its mechanisms of action and its potential role in advancing equitable perinatal mental health care.

### 8.1 Mechanisms and clinical interpretation

WET’s alignment with perinatal care extends beyond its brevity to its underlying mechanisms of trauma processing (36). By promoting repeated written engagement with trauma narratives, WET facilitates emotional processing and extinction learning, which directly target avoidance, hyperarousal, and intrusive symptoms that can interfere with caregiving and bonding. These mechanisms may be particularly salient during pregnancy and the postpartum period, where trauma symptoms often co-occur with ongoing physiological stressors, sleep disruption, and heightened caregiving demands. In contexts such as high-risk pregnancies or NICU admissions, where trauma exposure is prolonged or cumulative, WET’s structured, time-limited format may support symptom reduction without requiring extensive cognitive restructuring or between-session demands. Through this mechanism, WET has the potential not only to reduce PTSD symptoms but also to improve maternal functioning, engagement in care, and early relational processes that influence infant outcomes (28, 39, 46). Importantly, WET is not intended to directly address the structural determinants that generate systemic trauma. Rather, its value within a systemic trauma framework lies in mitigating the psychological consequences of trauma and reducing barriers to accessing evidence-based PTSD treatment within existing healthcare systems.

### 8.2 Equity and subgroup-specific implications

Positioning WET as an equity strategy requires attention to how systemic trauma disproportionately shapes risk and access to care across perinatal populations (22, 24). For example, Black women navigating obstetric racism and fragmented healthcare systems face both elevated PTSD risk and barriers to sustained mental health treatment, making low-burden, accessible interventions particularly critical. Similarly, parents of infants in the NICU experience sustained trauma exposure alongside logistical constraints such as extended hospital stays, transportation challenges, and caregiving demands that limit participation in traditional therapies. WET’s adaptability to telehealth and integration within obstetric and NICU-adjacent care pathways offers a mechanism to reduce these access barriers. Embedding WET within existing clinical touchpoints—rather than relying on referral-based outpatient models—supports more equitable access to trauma-focused care and aligns intervention delivery with the structural conditions in which perinatal PTSD develops.

### 8.3 Critical evaluation of current evidence

Several limitations of the current evidence base warrant careful consideration. First, there is a lack of large-scale randomized controlled trials evaluating WET specifically within perinatal populations, limiting causal inferences regarding effectiveness in these contexts (4, 15, 18). Second, existing studies are often conducted in controlled or high-resource environments and may be subject to selection bias, restricting generalizability across diverse perinatal populations and healthcare systems. Third, the broader literature remains disproportionately U.S.-centric, with limited data on perinatal PTSD and trauma-focused treatment delivery in low- and middle-resource settings (2). Although the proposed framework was developed primarily from evidence generated within U.S. healthcare systems, several core elements are likely relevant across international settings, including the role of trauma exposure during the perinatal period, the impact of PTSD on maternal and infant outcomes, and the need for accessible trauma-focused interventions. However, the specific structural drivers of systemic trauma, healthcare delivery models, insurance systems, workforce capacity, and pathways for integrating mental health services may differ considerably across countries and healthcare systems. As a result, implementation of the proposed framework will likely require adaptation to local healthcare infrastructure, cultural contexts, and available perinatal mental health resources. Finally, as this manuscript represents a conceptual synthesis rather than primary empirical research, the proposed framework is hypothesis-generating. Although it integrates existing theoretical and clinical evidence, its assumptions require validation through rigorous empirical study across diverse populations and care contexts.

### 8.4 Actionable future directions

Advancing WET within perinatal care systems requires coordinated, methodologically rigorous next steps that move beyond conceptual alignment to empirical testing and implementation (36, 37). First, early-phase and pilot clinical trials are needed to establish the feasibility, acceptability, and safety of delivering WET across the perinatal timeline, including during pregnancy, the postpartum period, and within NICU settings (20, 21). These studies should evaluate not only symptom reduction but also maternal functioning under conditions of medical complexity, sleep disruption, and fragmented care. Second, mechanism-focused research is needed to better understand how trauma-focused symptom change translates into improvements in maternal–infant bonding, emotion regulation during caregiving, and physiologic pathways linked to adverse pregnancy outcomes, such as inflammation and hypertensive disorders (8). Clarifying these mechanisms will strengthen the theoretical and clinical rationale for integrating WET into perinatal care.

Although existing evidence suggests that trauma-focused treatments can be delivered safely during the perinatal period, ongoing clinical monitoring and coordination with obstetric providers remain important, particularly in high-risk pregnancies or in the presence of significant psychiatric comorbidity. Future research should further evaluate safety, tolerability, clinical monitoring needs, and implementation considerations for WET across diverse perinatal populations. An important implementation consideration concerns the workforce required to deliver WET within perinatal settings. Although obstetric providers play a critical role in screening, referral, and care coordination, delivery of trauma-focused psychotherapy will likely be most feasible through integrated behavioral health teams or dedicated mental health professionals with training in evidence-based trauma treatments. Future implementation studies should evaluate the comparative feasibility, sustainability, and resource requirements of these delivery models across diverse healthcare settings.

Third, implementation-focused studies are critical to determining how WET can be embedded within obstetric, pediatric, NICU, and safety-net settings (37). This includes the development of scalable delivery models, fidelity monitoring procedures, and training pathways for clinicians working across disciplines. Integration within maternal health equity initiatives and alignment with existing obstetric workflows will be essential for sustainability (33, 34). Fourth, future research should prioritize subgroup-specific analyses to evaluate differential effects across populations disproportionately affected by systemic trauma, including racial and ethnic minority groups and those receiving care in low-resource settings. Such work is necessary to ensure that WET is not only effective but equitably delivered. Finally, qualitative and mixed-methods approaches should be used to capture patient and provider perspectives, refine intervention delivery, and support the co-design of adaptations that are responsive to the lived experiences of perinatal populations.

## 9 Conclusions

Perinatal posttraumatic stress disorder unfolds within a landscape shaped by systemic trauma, structural inequities, discriminatory care experiences, and chronic barriers to access, that amplify risk across pregnancy, childbirth, and the postpartum/NICU period. Within this context, untreated PTSD contributes to maternal morbidity, impaired bonding, and infant socioemotional vulnerability, with disproportionate burden among marginalized families. This perspective advances a systemic trauma-informed framework that reconceptualizes trauma-focused treatment as an embedded component of perinatal care, rather than a referral-based service. While the current evidence base remains limited, particularly in perinatal populations and globally diverse contexts, this framework offers a clinically actionable model for integrating brief, scalable interventions such as Written Exposure Therapy within existing care pathways. Advancing this agenda will require coordinated efforts across obstetrics, psychiatry, pediatrics, and implementation science, alongside rigorous evaluation in diverse populations and care settings. By aligning trauma-focused care with broader maternal health equity initiatives, this work has the potential to reduce disparities in access to evidence-based treatment, strengthen maternal–infant relationships, and interrupt the intergenerational transmission of trauma. Translating this framework into practice will require aligned efforts across policy, clinical training, and implementation infrastructure to ensure that trauma-focused care is accessible within routine perinatal settings.

## Data Availability

The original contributions presented in the study are included in the article/supplementary material. Further inquiries can be directed to the corresponding author.
